# Integrin Crosstalk and Its Effect on the Biological Functions of the Trabecular Meshwork/Schlemm’s Canal

**DOI:** 10.3389/fcell.2022.886702

**Published:** 2022-04-29

**Authors:** Jennifer A. Faralli, Mark S. Filla, Donna M. Peters

**Affiliations:** ^1^ Department of Pathology and Laboratory Medicine, University of Wisconsin School of Medicine and Public Health, Madison, WI, United States; ^2^ Department of Ophthalmology and Visual Sciences, University of Wisconsin School of Medicine and Public Health, Madison, WI, United States

**Keywords:** trabecular meshwork, glaucoma, integrin, extracelluar matix, crosstalk

## Abstract

Integrins are a family of heterodimeric receptors composed of an α- and β-subunit that mediate cell-adhesion to a number of extracellular matrix (ECM) proteins in the Trabecular Meshwork/Schlemm’s canal (TM/SC) of the eye. Upon binding an ECM ligand, integrins transmit signals that activate a number of signaling pathways responsible for regulating actin-mediated processes (i.e phagocytosis, cell contractility, and fibronectin fibrillogenesis) that play an important role in regulating intraocular pressure (IOP) and may be involved in glaucoma. An important function of integrin-mediated signaling events is that the activity of one integrin can affect the activity of other integrins in the same cell. This creates a crosstalk that allows TM/SC cells to respond to changes in the ECM presumably induced by the mechanical forces on the TM/SC, aging and disease. In this review, we discuss how integrin crosstalk influences the function of the human TM/SC pathway. In particular, we will discuss how different crosstalk pathways mediated by either the αvβ3 or α4β1 integrins can play opposing roles in the TM when active and therefore act as on/off switches to modulate the cytoskeleton-mediated processes that regulate the outflow of aqueous humor through the TM/SC.

## Introduction

The extracellular matrix (ECM) creates a microenvironment that influences gene expression and cellular behavior in tissues (Iozzo and Gubbiotti, 2018; Seetharaman and Etienne-Manneville, 2018). In the human eye, the composition and biomechanical properties (rigidity, elasticity and topology) of the ECM within the Trabecular Meshwork/Schlemm’s Canal (TM/SC) contribute to the regulation of intraocular pressure (IOP) and the pathogenesis of glaucoma (Keller and Peters, 2022). Changes in the ECM affect IOP by altering the biological processes (i.e contractility, gene expression, or phagocytosis) in the TM/SC that regulate aqueous humor flow through the anterior chamber (Rohen and Lutjen-Drecoll, 1989; Rohen et al., 1993; Vranka et al., 2015).

The TM/SC is a highly elastic tissue that stretches and recoils in response to mechanical forces. These mechanical forces are triggered by contraction of the ciliary muscle and pulsatile forces due to changes in IOP and blood pressure (Johnstone et al., 2021). Such changes in the TM/SC pathway are likely to cause force-induced changes in ECM fibrils like fibronectin (Baneyx et al., 2002; Smith et al., 2007) that can be detected by specific cell surface receptors, thus enabling the cells in the TM/SC to change their biological functions in response to mechanical and pulsatile forces.

Integrins are a major class of cell surface receptors that are able to respond to changes in the TM/SC ECM. They are a family of transmembrane heterodimeric proteins composed of an α and β subunit. Eight β subunits and eighteen α subunits can be assembled into 24 distinct integrins with unique properties (Humphries et al., 2006). The various combinations of the integrin subunits create a heterodimer that shows specificity for different ECM ligands. Currently, 12 different integrins have been identified on the cells in the TM/SC outflow pathway (Zhou et al., 1996; Zhou et al., 1999; Filla et al., 2017). Most of them appear to be expressed by all the cells in the TM/SC although there does appear to be some differences, most notably, in the levels of α2β1, α4β1 and αvβ3 integrin (Zhou et al., 1999). The expression profile and activity of these integrins in the TM/SC, however, are likely to vary. A number of factors such as the rigidity of the ECM (Seetharaman and Etienne-Manneville, 2018), ion channels and cadherins (Dieterle et al., 2021) and proteins associated with the cytoplasmic tails (Sun Z. et al., 2016) play a critical role in mediating integrin activity. In addition, since integrins are recycled on and off the cell surface and the mechanisms regulating this are cell type specific (Moreno-Layseca et al., 2019), it is possible that not all integrins will be expressed at the same time. Thus, the expression and mechanotransduction function of integrins will change in a spatiotemporal fashion as the physical and mechanical properties of the TM/SC varies.

Historically, integrins were thought to be cell adhesion receptors found in structures called focal adhesions (FAs). However, integrins are also found in other cellular structures (Zuidema et al., 2020) found in the TM/SC. For instance, α5β1 integrin is found in fibrillar adhesions. The α3β1 integrin and the α6β4 integrin can be found in cadherin-containing adherens junctions (AJs) and hemidesmosomes, respectively. Some integrins like α3β1 and αvβ3 are also found in invadiopodum/podosomes which can be found in TM cells (Aga et al., 2008; Murphy and Courtneidge, 2011; Zuidema et al., 2020) and recently the α3β1 integrin was found in tight junctions in the TM/SC (Li et al., 2020). Other integrins, such as αvβ5 integrin, can be found in clathrin-containing adhesion complexes whereas α3β1, α6β1, and α4β1 integrins can be found in tetraspanin-enriched microdomains (Zuidema et al., 2020). Thus, integrins have multiple roles in cells mediating attachment to the substrate, cell-cell adhesion, endocytosis, and the assembly of signaling and membrane complexes.

**FIGURE 1:**
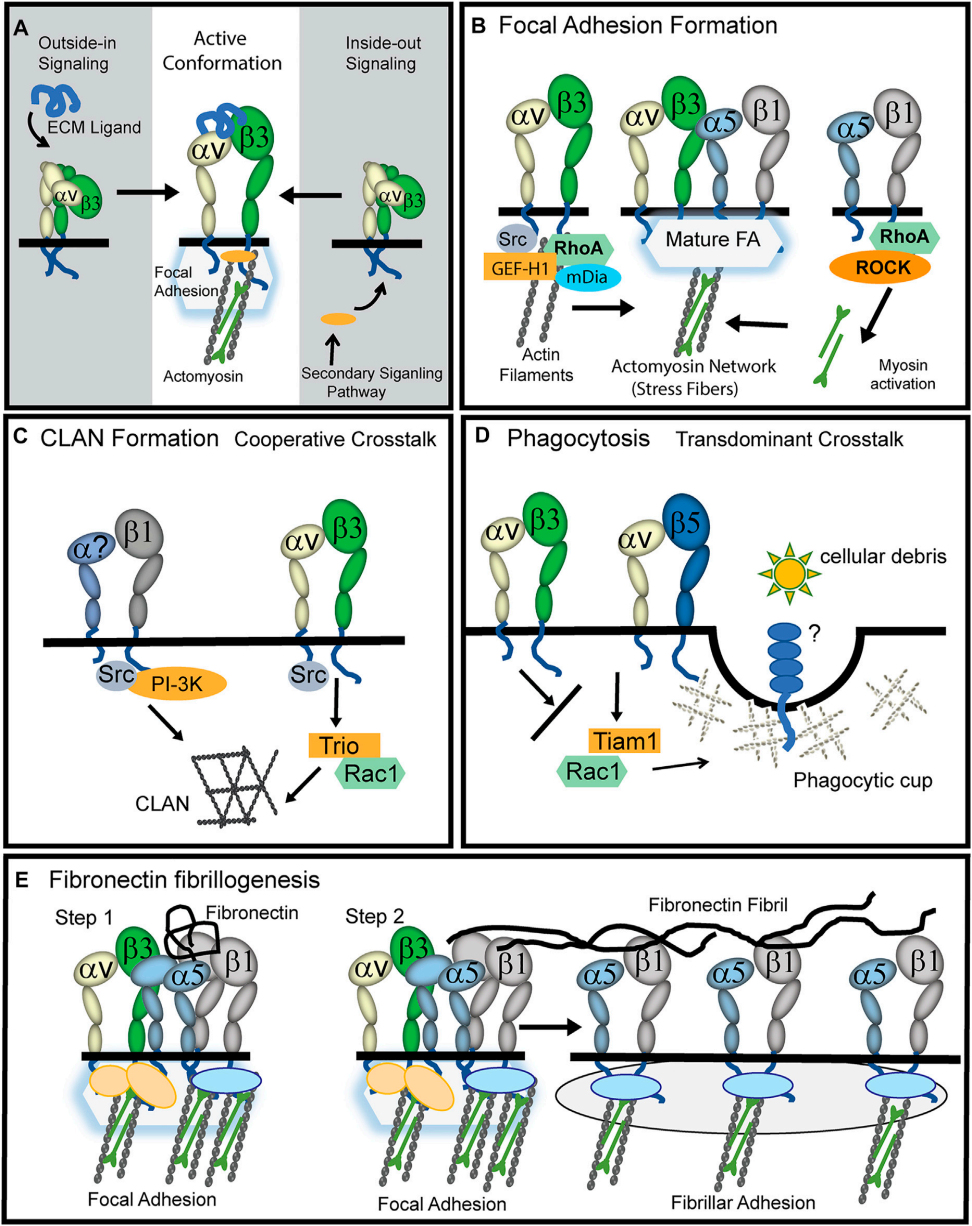
Role of activated αvβ3 integrin in TM cells. **(A)** The active conformation of αvβ3 integrin can be triggered by a process called outside-in signaling in which an ECM ligand binds to the extracellular domain of the heterodimer and triggers the upright conformation. Alternatively, a process called inside-out signaling can induce the active conformation. This happens when a secondary signaling pathway triggers the binding of cytoplasmic proteins (orange oval) to the cytoplasmic tails of the integrin causing the cytoplasmic tails to separate and the heterodimer to assume an upright conformation. **(B)** During the early stages of focal adhesion (FA) formation, actin filaments are generated when activated αvβ3 integrin signaling recruits GEF-H1, RhoA and the Rho effector mDía to nascent FAs. Stress fibers in FAs are then formed when signaling from α5β1 integrins recruit RhoA and Rho kinase (ROCK) to the FA and myosin is activated and binds to actin filaments. **(C)** Cooperative crosstalk between activated αvβ3 and β1 integrins forms CLANs. αvβ3 integrins utilize Src kinase to help recruit the GEF Trio and Rac1 to FAs while β1 integrins utilize Src kinase and PI3-kinase. The question mark in the α subunit indicates that α1, α2, α4, or α5 integrin subunits may be involved. **(D)** Transdominant crosstalk between αvβ3 and αvβ5 integrins inhibits αvβ5 integrin-mediated phagocytosis which involves the GEF Tiam1 and Rac1. αvβ3 integrin inhibits this process presumably because it prevents αvβ5 integrin from using Rac1 to form the branched actin structure used to form a phagocytic cup. **(E)** Upon binding of soluble fibronectin to α5β1 integrins (Step1), cooperative signaling between αvβ3 and α5β1 integrins in FAs creates the RhoA-mediated contractility needed for fibronectin fibrillogenesis (Zhang et al., 1997; Zhong et al., 1998). While αvβ3 integrins remain in FAs, presumably to help anchor stress fibers, α5β1 integrins are translocated out of FAs by contractile forces into fibrillar adhesions (Step2). This α5β1 translocation promotes the stretching of the fibronectin dimer which exposes fibronectin-fibronectin binding sites involved in fibronectin fibrillogenesis.

A unique feature of integrins is that their activity involves very specific conformational changes in their α- and β-subunits (Askari et al., 2009; Campbell and Humphries, 2011; Li et al., 2017; Vicente-Manzanares and Sanchez-Madrid, 2018; Kechagia et al., 2019). In their unoccupied state, the extracellular domains of the α and β integrin subunits are in a bent conformation with their cytoplasmic tails bound together by a salt bridge (Figure 1A). Upon activation, the integrin subunits undergo a conformational change and assume an upright conformation with their cytoplasmic tails separated. The integrin can be activated by engaging its ligand. This is called outside-in signaling. Alternatively, the integrin can be activated intracellularly by a secondary signaling pathway which allows the integrin to engage its ligand. This is called inside-out signaling.

Upon engagement with an ECM protein, each integrin transmits signals *via* proteins associated with their cytoplasmic tails and the cytoskeleton. This cytoskeleton engagement allows for signals generated by mechanical forces on the ECM to be transmitted directly to the nucleus and drive transcription. Thus, integrins are conduits that convert signals from the ECM environment (rigidity, elasticity and topology) into intracellular biochemical signals that affect cytoskeleton organization, gene expression, and proliferation (Kechagia et al., 2019).

The specificity and activation of each integrin can occur within subseconds and is therefore likely to be highly regulated by the spatiotemporal expression and activation of other integrins and receptors on the cell surface (Ross et al., 2013; Seetharaman and Etienne-Manneville, 2018). This creates not only a “crosstalk” between integrins but a crosstalk between integrins and their various membrane partners such as cadherins (Canel et al., 2013), syndecans (Bass and Humphries, 2002; Morgan et al., 2007), and growth factor receptors (Ross, 2004). This crosstalk, together with the specific conformational states of the integrin, enables the integrin function to be tunable thus eliciting different biochemical responses without necessarily disrupting cell adhesion.

At least two integrins found in the TM/SC are known to have a “tunable” conformation and thus their activity is likely to be altered depending upon the composition and 3D architecture of the ECM in the TM/SC. These integrins are the αvβ3 and α4β1 integrins.

### αvβ3 Integrin

αvβ3 integrin is potentially a key player in regulating outflow through the TM/SC and is found throughout the TM/SC (Gagen et al., 2013; Filla et al., 2017). Unlike other integrins in this pathway, αvβ3 integrin has a number of ligands important in regulating IOP including connective tissue growth factor (CTGF), fibronectin, and thrombospondin-1 (TSP-1). Its expression and active state can be upregulated by the glucocorticoid dexamethasone (Filla et al., 2011; Faralli et al., 2013) *via* a secondary effect involving the transcription factor NFATc1. CTGF may also upregulate αvβ3 integrin activity since it increases expression of the αv integrin subunit in human TM (HTM) cells (Junglass et al., 2009). αvβ3 integrin can also be activated by mechanical forces similar to those observed in the TM/SC (Johnstone et al., 2021). In particular, the αvβ3 integrin can be activated by shear stress (Tzima et al., 2001) making it likely to be activated on SC cells when shear stress is increased (Ashpole et al., 2014).

Transforming growth factor β2 (TGFβ2), which is a risk factor for primary open angle glaucoma (POAG) (Fuchshofer and Tamm, 2012), increases expression of both αv and β3 integrin subunits (Tsukamoto et al., 2018). Interestingly, αvβ3 integrin activation drives expression of TGFβ2 (Filla et al., 2021), thus setting up a potential feedback loop. Additionally, TGFβ2-induced changes in the ECM (Fleenor et al., 2006; Medina-Ortiz et al., 2013) likely trigger the expression of various ECM ligands (Flugel-Koch et al., 2004) that can activate αvβ3 integrin. For instance, TSP-1, which is upregulated by TGFβ2, can activate αvβ3 integrin signaling (Gao et al., 1996; Barazi et al., 2002; Filla et al., 2009).

αvβ3 integrins likely play a role in mechanosensing in the TM/SC pathway. Studies in fibroblasts found that integrins containing the αv integrin subunit in FAs play an important role in modulating cellular responses to forces on the ECM microenvironment (Schiller et al., 2013). αvβ3 integrin localization into FAs is driven by forces on the actomyosin network (Wang and Ha, 2013), and it is considered to be one of the more stable components of FAs (Morgan et al., 2013). This is in contrast to α5β1 integrin which ultimately leaves FAs and translocates to fibrillar adhesions (Katz et al., 2000). αvβ3 integrin is, therefore, a key factor in the mechanosensing role of FAs and its localization in FAs is a prerequisite for myofibroblast differentiation (Hinz, 2006; Hinz and Gabbiani, 2010; Hinz et al., 2012) which is thought to drive ECM changes associated with POAG (Fuchshofer and Tamm, 2012).

Another important feature of αvβ3 integrin is that it has a weaker bond strength and faster binding and unbinding rate compared to α5β1 integrin within FAs (Roca-Cusachs et al., 2009). This makes αvβ3 integrin better able to sense and modulate changes in the contractile properties of cells as the TM/SC stretches and recoils, possibly acting as an on/off switch to control the contractile properties of the TM/SC cytoskeleton. This presumably would involve force-induced changes in the interactions between αvβ3 integrin and its ECM ligands (Seetharaman and Etienne-Manneville, 2018) that may occur as the TM/SC stretches and recoils. Other factors that may affect mechanosensing through integrins in the TM/SC include lipid rafts (Radel et al., 2007; Sun X. et al., 2016), cross talk with ion channels and cadherins (Dieterle et al., 2021) and the mechanical link (clutch bond) formed between integrins and actin binding proteins (Sun Z. et al., 2016).

**FIGURE 2:**
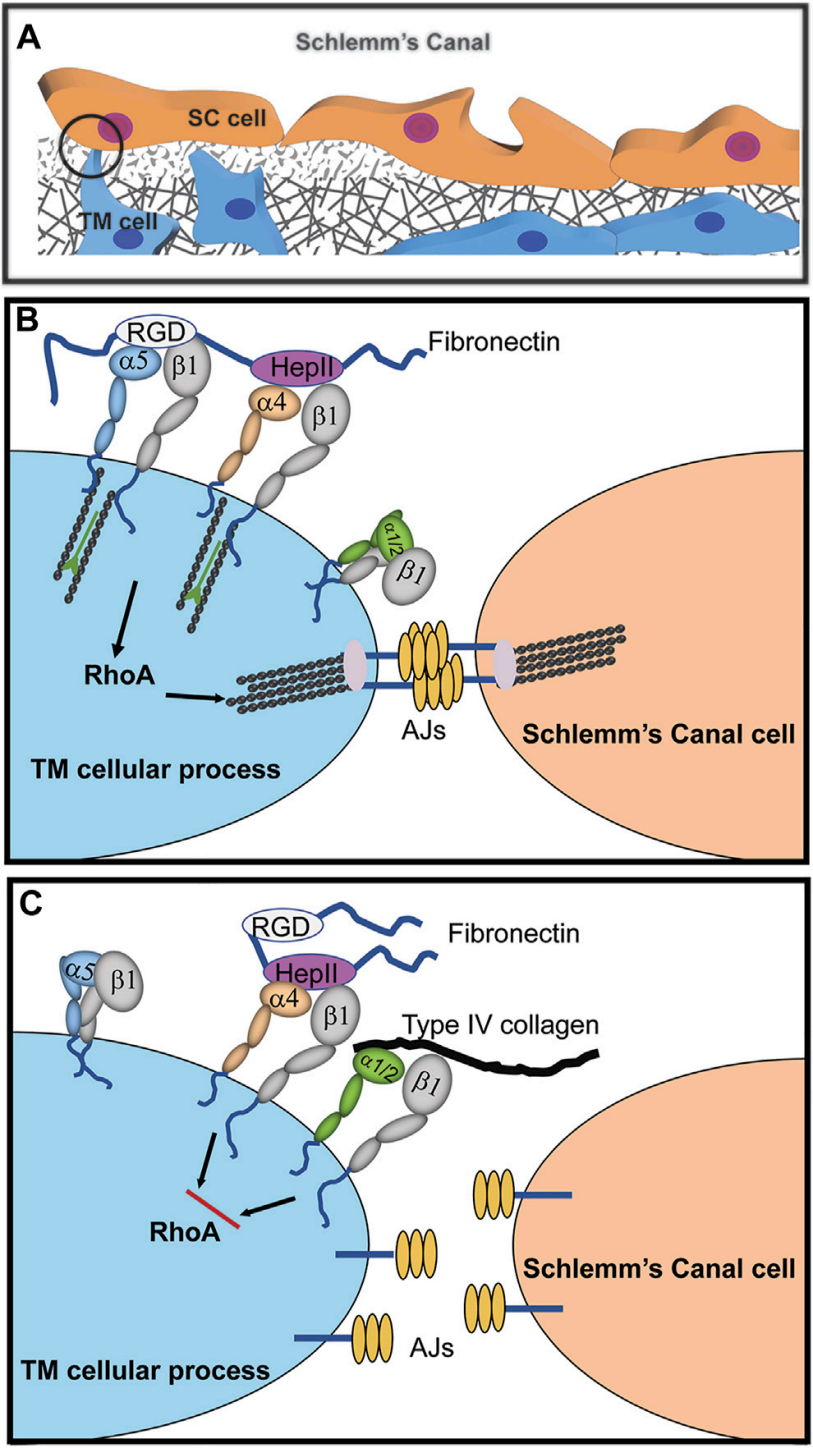
Cooperative signaling with α4β1 integrin controls assembly of adherens junctions (AJs). **(A)** Cadherin-containing AJs are likely to be found in the cellular processes connecting juxtacanalicular trabecular meshwork (JCT) and SC cells as well as in cellular processes connecting JCT cells to each other and with JCT cells connected to the cells lining the trabecular beams of the meshwork (Johnstone et al., 2021). **(B)** Fibronectin in the ECM promotes cooperative signaling between α4β1 and α5β1 integrins that activates RhoA-mediated stress fiber formation. Crosstalk between this integrin signaling complex and cadherins in AJs stabilizes cell-cell interactions. **(C)** The presence of collagen in the ECM binding to either α1β1 or α2β1 integrins disrupts α4β1 integrin signaling with α5β1 integrin. This creates an α1/α2β1 and α4β1 integrin complex that inhibits RhoA activity, thus causing a disruption in the formation of stress fibers and AJs.

αvβ3 integrin is responsible for regulating a number of different actin-containing structures. It is responsible for the Src/Rac1 driven polymerization of branched Arp2/3-containing actin networks in lamellipodium (Danen et al., 2002) and crosslinked actin networks (CLANs) in HTM cells (Filla et al., 2009). It is also involved in RhoA mediated formation of podosomes in osteoclasts (Chellaiah, 2006) and the RhoA-mDia polymerization of actin filaments during early stages of cell adhesion (Figure 2A). Eventually, these actin filaments formed by αvβ3 integrin-RhoA-mDia signaling develop into stress fibers in mature FAs when RhoA/ROCK activity generated by α5β1 integrin drives the phosphorylation of myosin (Huveneers and Danen, 2009).

In the following section we will discuss what is known about how αvβ3 integrin crosstalk influences the formation of various actin-containing structures required for phagocytosis, fibronectin fibrillogenesis and CLAN formation in the TM/SC pathway. In each case, the role of αvβ3 integrin appears to be determined by the guanine nucleotide exchange factors (GEFs) recruited into the integrin signaling complex.

#### Role of αvβ3 Integrin in Phagocytosis

*In vitro* studies show that αvβ3 integrin triggers Rac1-mediated signaling events in HTM cells associated with glaucoma. For instance, activation of αvβ3 integrin decreased phagocytosis (Gagen et al., 2013; Peotter et al., 2016). Phagocytosis is needed to clear cellular debris from the outflow pathway, and dysregulation of phagocytosis is associated with elevated IOP in glucocorticoid-induced glaucoma (Matsumoto and Johnson, 1997a; Matsumoto and Johnson, 1997b). Normally, an αvβ5 integrin driven Rac1 process regulates phagocytosis in HTM cells (Gagen et al., 2013) as observed in retinal pigmented epithelial cells (Mao and Finnemann, 2012). The inhibition of phagocytosis by αvβ3 integrin activation is considered a “transdominant inhibition” (Diaz-Gonzalez et al., 1996; Gonzalez et al., 2010) of αvβ5 integrin activity (Figure 1D). Studies suggest the inhibition was due to a switch in the use of the GEFs (Trio or Tiam1) regulating the activation of Rac1 (Peotter et al., 2016; Faralli et al., 2019a) since αvβ5 integrin expression was unimpaired by upregulation of an activated αvβ3 integrin (Gagen et al., 2013).

#### Role of αvβ3 Integrin in Fibronectin Fibrillogenesis

αvβ3 integrin activation in cultured HTM cells increased the deposition of the alternatively spliced form of fibronectin called EDA + fibronectin (Filla et al., 2019) that is associated with the ECM changes observed in POAG and thought to contribute to elevated IOP (Roberts et al., 2020). This enhanced EDA + fibronectin fibrillogenesis may be due to cooperativity between αvβ3 integrin-Rac1 mediated actin polymerization and the α5β1 integrin/Rho signaling pathway forming stable FAs that promote the RhoA-mediated contractile forces needed for fibrillogenesis (Figure 1E). Thus, during fibrillogenesis, αvβ3 integrin maintains the FAs, while the contractile forces of the actomyosin fibers drive α5β1 integrin translocation into fibrillar adhesions; pulling and stretching the fibronectin as they form fibrils.

#### Role of αvβ3 Integrin in CLAN Formation

αvβ3 integrin is also involved in CLAN formation (Figure 1C). CLANs are interconnected networks of actin filaments that radiate outward from central hubs resembling a geodesic dome (Lazarides, 1976). CLANs were found in dexamethasone treated-cultured TM cells and in TM cells in isolated meshworks from glaucomatous donor eyes not treated with dexamethasone (Clark et al., 1995) suggesting these actin structures are involved in POAG pathogenesis (Clark et al., 2005; Hoare et al., 2009). The αvβ3 integrin is suitable to controlling CLAN formation since it readily reorganizes the actin cytoskeleton in response to lower levels of stiffness in the ECM like that induced by stretch (Balcioglu et al., 2015). The function of CLANs in the TM remains unclear, but they were originally thought to be actin stress fiber precursors in nonmuscle cells (Lazarides, 1976). If true, this suggests that increased CLAN formation could trigger increased stress fiber formation which may contribute to elevated IOP in glaucoma (Rao et al., 2001; Rao et al., 2005).

CLAN assembly, like fibronectin fibrillogenesis, also involves cooperativity between αvβ3 integrin and a β1 integrin and is dependent on the level of αvβ3 integrin expression and activity (Filla et al., 2009). However, unlike fibronectin fibrillogenesis, this crosstalk between αvβ3 and β1 integrins utilizes a Rac1-mediated signaling pathway to control actin polymerization (Figure 1D). This difference is likely due to the downstream effectors being used to activate actin polymerization. Studies in HTM cells showed that during CLAN formation, αvβ3 integrin signaling activates the Rac1 GEF Trio (Filla et al., 2009). This αvβ3 integrin/Trio/Rac1 signaling pathway converges with a β1 integrin/PI3-K signaling pathway to form CLANs. Both β1 and αvβ3 integrins are needed since low levels of CLANs are observed in HTM cells when only one of these integrins is engaged (Filla et al., 2006). Although α5β1 and αvβ3 integrins were more proficient at inducing CLANs, other β1 integrins such as α4β1 integrin and the collagen-binding integrins α1β1 and α2β1 induce CLANs in cooperation with αvβ3 integrins (Filla et al., 2006). Thus, the frequency of CLAN formation is dependent on ECM substrate composition and the engagement of specific integrins with the ECM.

Given the effects of αvβ3 integrin on phagocytosis, ECM deposition and actin polymerization, it is not surprising that αvβ3 integrin activation significantly increased IOP in C57BL/6J mice while the knockdown of αvβ3 integrin levels in the mouse TM significantly decreased IOP (Faralli et al., 2019b). αvβ3 integrin activation also significantly decreased outflow facility in porcine cultured anterior segments (Faralli et al., 2019b).

### α4β1 Integrin

α4β1 is another integrin found in the TM/SC pathway (Zhou et al., 1999; Peterson et al., 2004) that has a well-documented tunable function (Chigaev et al., 2001; Chigaev et al., 2004; Chigaev et al., 2007). Although it is best known for its role in inflammation as a co-receptor for TLR4 (Kelsh-Lasher et al., 2017) and as an adhesion receptor in migrating leukocytes (Liu et al., 2000), α4β1 integrin also controls the shear force-dependent contractile properties of endothelial cells (Goldfinger et al., 2008) and the contractile properties of migrating smooth muscle and neural crests cells (Sheppard et al., 1994; Stepp et al., 1994). Among the integrins, it has a unique role in migration and FAs that is largely attributed to the α4 integrin cytoplasmic domain (Kassner et al., 1995). α4β1 integrin forms less stable interactions with the cytoskeleton within FAs similar to αvβ3 integrin which suggests that it may alter cytoskeletal dynamics in response to mechanical factors such as shear force (Goldfinger et al., 2008). This weaker interaction is regulated by paxillin binding to the α4 subunit cytoplasmic tail. When paxillin is bound to α4β1 integrin, binding to the α4β1 ligand is weakened (Liu et al., 2002). Paxillin interactions with the α4 subunit are regulated by the phosphorylation of Ser^988^ in the α4 cytoplasmic tail (Han et al., 2001). When Ser^988^ is phosphorylated, interactions between paxillin and the α4 cytoplasmic tail are inhibited.

Like αvβ3 integrin, α4β1 integrin can be activated by inside-out signaling pathways (Figure 1A). These pathways include G-protein-coupled receptors for CXCR2 and CXCR4 (Laudanna et al., 2002), phorbol esters, and calcium ionophores (Chigaev et al., 2007). In contrast, other signaling pathways such as nitric oxide/cGMP pathways in lymphoma cells can downregulate α4β1 integrin activation (Chigaev et al., 2011).

One α4β1 integrin ligand is the HeparinII (HepII) domain of fibronectin (Faralli et al., 2019c) Studies using the HepII domain found that α4β1 integrin can control the contractile properties of the TM/SC. Treating HTM cultures with the HepII domain caused the disassembly of stress fibers and decreased cell contractility in a collagen gel assay (Schwinn et al., 2010). This required the α4β1 integrin because when α4β1 integrin expression was silenced, the HepII domain had no effect on cell contractility. Perfusion of the HepII domain also decreased IOP in human (Santas et al., 2003) and monkey cultured anterior segments (Schwinn et al., 2010). Histology of the monkey anterior segments showed that treatment with the HepII domain disrupted the contractile properties of the TM/SC similar to what was observed in monkey eyes treated with the H-7 inhibitor (Sabanay et al., 2000). Additionally, there was a loss of SC cells in human anterior segments (Santas et al., 2003) possibly resulting from a disruption in cell-cell adhesions since HepII treatment disrupted AJs in HTM cultures (Gonzalez et al., 2006; Gonzalez et al., 2009). These data suggest that α4β1 integrin activation by the HepII domain affected the contractile properties of the TM/SC.

The composition of the ECM can affect the ability of the HepII domain and α4β1 integrin to disrupt the contractile properties of HTM cells (Figure 2). When TM cells were plated on type IV collagen, not only was stress fiber assembly disrupted by the HepII domain, but AJs were also disrupted (Peterson et al., 2004; Schwinn et al., 2010). Presumably this loss in stress fibers and AJs occurred because RhoA, which is needed to stabilize AJs and stress fibers, was inhibited. In contrast, when cells were plated on fibronectin, HepII domain enhanced stress fiber formation and AJs were unaffected. Since different integrins would be involved in binding to fibronectin versus type IV collagen, this suggests that crosstalk between the various integrins and α4β1 integrin differentially affect RhoA. Crosstalk between collagen-bound integrins (α1β1 or α2β1) and HepII-bound α4β1 integrin may disrupt RhoA activity whereas crosstalk between the α5β1 integrin fibronectin receptor and HepII-bound α4β1 integrin enhance RhoA activity (Peterson et al., 2004; Schwinn et al., 2010). This observation has special significance for the TM/SC because changes in ECM composition, especially an increase in fibronectin expression, are associated with increased IOP and the development of POAG. Thus, this finding demonstrates how critical integrin-ECM interactions are for modulating the functions of the TM/SC.

## Discussion

Since the expression of and signaling from αvβ3 and α4β1 integrins (Peterson et al., 2004; Faralli et al., 2013) is normally low in HTM cells *in vitro* compared to other integrins, activation of these two integrins could be a gain of function in normal HTM cells. Interestingly, activating αvβ3 integrin as discussed above would induce many of the changes associated with reduced aqueous humor outflow while activating α4β1 integrin in TM increases outflow facility. This suggests that the level of expression and activity of these two integrins could essentially create on/off switches in the TM/SC pathway that could regulate the homeostatic properties of the tissue.
